# Connectedness of healthcare professionals involved in the treatment of patients with Parkinson's disease: a social networks study

**DOI:** 10.1186/1748-5908-6-67

**Published:** 2011-07-03

**Authors:** Michel Wensing, Martijn van der Eijk, Jan Koetsenruijter, Bastiaan R Bloem, Marten Munneke, Marjan Faber

**Affiliations:** 1 Scientific Institute for Quality of Healthcare (IQ healthcare), Radboud University Nijmegen Medical Centre, P.O. Box 9101, 6500 HB Nijmegen, Nijmegen, Netherlands; 2 Department of Neurology, Donders Institute for Brain, Cognition and Behaviour, Radboud University Nijmegen Medical Centre, P.O. Box 9101, 6500 HB Nijmegen, Nijmegen, Netherlands

## Abstract

**Background:**

Patients with chronic illness typically receive ambulatory treatment from multiple health professionals. Connectedness between these professionals may influence their clinical decisions and the coordination of patient care. We aimed to describe and analyze connectedness in a regional network of health professionals involved in ambulatory treatment of patients with Parkinson's disease (PD).

**Methods:**

Observational study with 104 health professionals who had joined a newly established network (ParkinsonNet) were asked to complete a pre-structured form to report on their professional contacts with others in the network. Using social networks methods, network measures were calculated for the total network and for the networks of individual health professionals. We planned to test differences between subgroups of health professionals regarding 12 network measures, using a random permutation method.

**Results:**

Ninety-six health professionals (92%) provided data on 101 professionals. The reciprocity of reported connections was 0.42 in the network of professional contacts. Measures characterizing the individual networks showed a wide variation; *e.g*., density varied between 0 and 100% (mean value 28.4%). Health professionals with ≥10 PD patients had higher values on 7 out of 12 network measures compare to those with < 10 PD patients (size, number of connections, two step reach, indegree centrality, outdegree centrality, inreach centrality, betweenness centrality). Primary care professionals had lower values on 11 out of 12 network measures (all but reach efficiency) compared to professionals who were affiliated with a hospital.

**Conclusions:**

Our measure of professional connectedness proved to be feasible in a regional disease-specific network of health professionals. Network measures describing patterns in the professional contacts showed relevant variation across professionals. A higher caseload and an affiliation with a hospital were associated with stronger connectedness with other health professionals.

## Background

Many patients with chronic diseases receive ambulatory treatment from a range of health professionals. Teamwork improves clinical performance, outcomes, and efficiency of healthcare [1]. Potential elements of good teamwork include improved coordination of care and integration of a wider range of professional competencies [2]. Contacts between health professionals are crucial in chronic illness care [3]. In primary and ambulatory care settings, where most chronic illness care is provided, health professionals have limited face-to-face contact with each other because most are based in office-based practices. In this situation, clinical processes and outcomes are determined by distributed decision making, involving many health professionals who may or may not share clinical knowledge and coordinate treatment delivery. It remains unclear how connectedness between health professionals influence ambulatory treatment.

Parkinson's disease (PD) provides an example of a chronic disease, which is largely treated in ambulatory care settings. PD is a common and progressive neurodegenerative disorder, which features both cognitive and motor symptoms [4]. The prevalence of PD is 1.6% in the Dutch population, with values increasing with age up to 4.3% in individuals aged 85 years or over [5]. PD cannot be cured, but pharmacological treatment substantially improves quality of life and functional capacity [4]. In addition, many patients require allied health care, including physical therapy, speech language therapy, and occupational therapy [6]. Thus, optimal treatment of PD requires a coordinated, multidisciplinary approach over a long period of time and implementation of recommended treatments [7].

To optimize multidisciplinary treatment, the ParkinsonNet concept has been developed: a professional regional network within the catchment area of hospitals [8,9]. ParkinsonNet aims to enhance PD-specific expertise among allied health providers by training a selected number of therapists according to evidence-based guidelines; by enhancing the accuracy of referrals to allied health workers by neurologists; by increasing patient volumes per therapist via preferred referral to ParkinsonNet therapists; and by stimulating collaboration between therapists, neurologists, specialized nurse practitioners, and patients [10]. ParkinsonNet is a regional network of a selected number of motivated health professionals with specific expertise in treating PD patients. The multidisciplinary networks are composed of a small number of highly motivated health care providers. Central to the ParkinsonNet concept are: delivery of care according to evidence-based guidelines; continuous education and training of ParkinsonNet health care providers; structured and 'preferred' referral to ParkinsonNet therapists by neurologists, enabling each therapist to attract a sufficient number of patients to maintain and increase expertise; optimal communication within the network via the internet, Meanwhile, more than 65 regional ParkinsonNet networks have been created in The Netherlands, now providing full nationwide coverage, with over 1,500 specialty-trained health care providers providing services. A cluster randomized trial showed that implementation of ParkinsonNet networks improved the efficiency of healthcare provision compared to usual care, at substantially reduced costs, while health outcomes remained unchanged [11].

Patterns in the professional contacts of health professionals involved in ParkinsonNet may influence clinical processes and outcomes in several ways. Specifically, professional contacts may improve the competence of health professionals regarding treatment of PD. Higher professional competence is associated with better clinical performance, quicker uptake of recommended interventions, and better outcomes for patients. It has been proposed that for most individuals, diffusion of innovations occurs through personal communication rather than through formal education or externally imposed sanctions [12]. Specific individuals (sometimes called 'knowledge brokers') may be crucial for introducing new ideas into a network. It seems reasonable to assume that professional competence regarding treatment of PD is highest in health professionals who treat ≥10 PD patients and in those affiliated with a specialized hospital department. Thus, connectedness with those two types of health professionals is expected to contribute to the spread of competence among health professionals in the network.

Connectedness between health professionals may also influence the coordination of patient care in treatment of PD. Better coordination may be associated with improved patient satisfaction and reduced health utilization, including less hospitalizations and fewer emergency visits [13]. In the absence of a strong formal organization and formalized leadership in a regional ParkinsonNet network, coordination of patient care is the result of informal social processes, which are characterized by distributed decision making. An example of such processes is the pressure on individuals who are embedded in highly connected networks to conform with the attitudes and behaviors of others in the network [14]. Also, individuals tend to link to similar others, resulting in networks with individuals who have similar attitudes and behaviors. We expected that health professionals would be more embedded in geographically defined catchment areas of specific hospitals than in the ParkinsonNet network in a region, if this includes more than one hospital.

Furthermore, network studies can identify informal leaders or highly influential individuals, who do not necessarily have a formalized leadership position. From a network perspective, these individuals are characterized by a specific position in the network, which gives them high social capital, *i.e*., control over connections [14]. It can be assumed that health professionals affiliated with a hospital have a central role in the treatment of PD, because they typically refer patients to other professionals. Thus, we hypothesized that primary care professionals would be less embedded in the network, most notably with respect to their prominence and influence in the network.

The aim of this study was to examine the connectedness in a newly established regional ParkinsonNet of health professionals involved in the treatment of PD patients. Our objectives were to examine the feasibility of a new measure; to describe the network in terms of a number of measures, which may be related to coordination of patient care and the spread of professional competence; and to examine the networks of health professionals with ≥10 PD patients and in those affiliated with a hospital.

## Methods

### Study design and population

We performed an observational study involving 104 health professionals in one specific region of 'ParkinsonNet' in the eastern part of The Netherlands. This network had been newly established a few weeks before the study was performed. The region has three hospitals, serving 600,000 inhabitants. Participants in the study were practicing health professionals from various medical, nursing, and allied health professions, who were based in either hospital settings or primary care. The medical ethical committee for Arnhem-Nijmegen approved the study.

### Measures

All 104 participants were requested to complete a structured questionnaire during an educational meeting, which was organized in the context of the network start-up; an email reminder was sent to non-responders. The questionnaire (which is available on request) listed all names of the health professionals in the network. Participants were asked to tick a box for each name indicating whether this person was known to the participant and another box to indicate whether this person was involved in professional contacts so far. Knowing each other was defined in the questionnaire as 'knowing the face, having talked to each with other, or having heard of.' Having professional contact was defined in the questionnaire as 'having had professional contact about at least one patient with PD who you are treating (including referral letters, emails, telephone contact, team meetings).' In addition, the questionnaire contained questions regarding health profession, number of patients with PD treated in one year (dichotomized into less than 10 versus 10 or more patients) as a measure for experience, and geographical location in the region (three hospital catchment areas were identified).

### Data analysis

Data were entered into a squared data-matrix with the health professionals in the rows and columns and values in the cells to indicate presence or absence of a connection (values 1 and 0, respectively). As a first step we examined the data with respect to missing scores, following published guidelines [15]. We examined the reciprocity of reported connections as an indicator of the reliability of the data collection instrument. Then we replaced missing values of the non-responders with the values provided by other individuals on the connection, if available. If no substitution was possible, the missing value was replaced with a zero. Missing values regarding individual characteristics were not substituted, except that we imputed a value for neurologists and specialized Parkinson nurses indicating that they treated more than 10 patients with PD.

The first stage of data analysis focused on the total network and the area-specific networks. Eight network measures were calculated for the networks of 'knowing each other' and 'having professional contact'. These network characteristics were expected to be relevant for professional competence and coordination of healthcare. The second stage of data analysis focused on the networks of the individual health professionals ('ego networks'). These individual networks were extracted from the total network for each health professionals, including the reported connections of the individual with others in the network and the connections between those others. Twelve measures were calculated for these individual networks, which were expected to be relevant for care coordination and professional competence.

Next, we explored the differences regarding the 12 measures of individual networks between subgroups of health professionals as defined by experience in treatment of patients PD (< 10 versus ≥10 PD patients, *i.e*., relatively little experienced versus much experience) and clinical setting (primary care versus hospital care or both). The cut-off level of 10 patients was based on consensus among the clinical authors of this paper. We hypothesized that health professionals treating many Parkinson patients and health professionals in specialized hospital settings would have higher values on the listed network characteristics. A random permutation test (with 10,000 permutations) was used to derive test differences between subgroups statistically. A p-value of 0.05 or less was considered significant. We used Excel to store and manage data files and UCINET 6 for descriptions and statistical analysis.

Finally, we performed an explorative factor analysis (principal component analysis with orthogonal rotation) on the 12 measures of individual networks to explore the correlational structure of the network measures. SPSS version 16 was used for this factor analysis.

## Results

A total of 96 of the 104 health professionals provided information on their connections (92%): 89 during the regional educational meeting, and seven after the email reminder (Table 1). Non-responders included one neurologist, one dietician, two occupational therapists, and four physiotherapists. Table 1 provides descriptive information on the sample. Ten different disciplines were represented in the regional network, with 44 physiotherapists comprising the largest group. About one-third (n = 35) worked in primary care and about one-half (n = 51) in both primary care and hospital settings. The remainder (n = 17) worked only in hospital. Less than one-half of the professionals (n = 43) treated more than 10 patients with PD. We found that the reciprocity of connections (before imputation of missing values and excluding mutually non-existent connections) was reasonably high: 0.57 in the network of 'knowing each other' and 0.42 in the network of 'professional contact.'

**Table 1:** Description of health professionals (n = 101)

	N
Professional background	
-neurologist (N)	3
-community geriatrician (O)	1
-specialized Parkinson nurse (V)	4
-dietician (D)	8
-occupational therapist (E)	20
-social worker (M)	1
-spiritual counselor (G)	1
-physiotherapist (F)	44
-psychologist (P)	3
-logopedic therapist (L)	16

Setting of care delivery	
Working in primary care	35
Working in hospital	17
Working in primary and hospital care	51

> 10 PD patients under treatment	43

Area	
1	28
2	32
3	41

**Figure 1:**
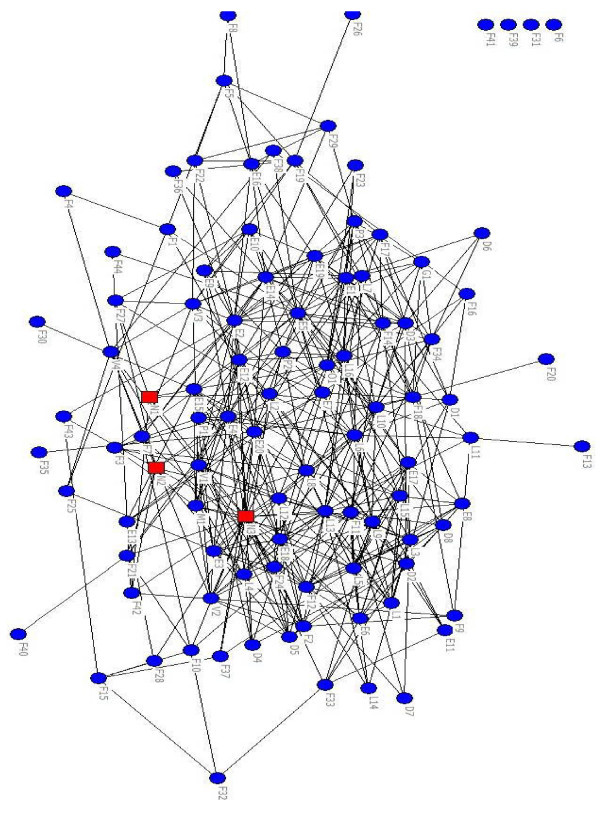
**Visual display of the total network of health professionals in ParkinsonNet**. Legend: health professionals with ≥10 PD patients in red, those with < 10 PD patients in blue.

Figure 1 presents the total network of connections between health professionals. Table 2 presents network characteristics of the total and area-specific networks, after imputation of missing values. The network of 'knowing each other' included more connections than the network of 'having professional contact' (1,431 versus 664). All other network measures also yielded higher values in the network of 'knowing each other.' Areas one and three showed higher values for network measures compared to the total network of professional contacts. The measures for area two showed a mixed picture: some were higher, others lower than in the total network. Area one had a relatively high outdegree centralization (33.7%), which suggests that a few health professionals were highly influential.

**Table 2:** Description of total and regional networks

	Knowing each other	Having professional contact			
	**Total network**	**Total network**	**Area one**	**Area two**	**Area three**

Number of health professionals	101	101	28	32	41

Total number of connections (ties)	1,431	664	113	91	158

Reciprocity	0.630	0.479	0.614	0.400	0.547

Density	0.142	0.066	0.139	0.092	0.146

Clustering (weighted)	0.360	0.268	0.344	0.253	0.395

Transitivity (three legs in triads with two legs)	16.7%	13.3%	16.9%	12.4%	20.9%

Indegree centralization of network	25.1%	16.6%	22.6%	10.5%	26.5%

Outdegree centralization of network	22.1%	16.6%	33.7%	27.2%	19.2%

Reciprocity: Proportion of all connections that are reciprocated. The measure is used as an indicator of the reliability of the measurement of connections.

Density: Proportion of all possible connections that are actually present in a network of a given size.

Clustering: Average density in the local neighborhoods of individuals rather than in the total network. Here it is defined as the density in the networks of others connected to an individual (leaving out ego in the calculation of density). The average value is weighted for size of network.

Transitivity: Measure related to triads that may indicate balance or equilibrium. If A directs a tie to B and B directs a tie to C, then A is also expected to direct to C. Triads are crucial in some social science theories.

Centralization of network: Degree of variance of the total network of (in/out going) connections compared to a perfect star network of the same size (which indicates the theoretical maximum of centralization). Higher values mean more centralization, thus that positional advantages are unequally distributed.

Table 3 shows a substantial variation of individual network characteristics for all measures in both the network of 'knowing each other' and in the network of 'having professional contact.' For example, the number of others known to the individual varied between 4 and 40, and the number of others in this network who can be reached in two steps varied between 36 and 99 (in those with at least one connection). Consistent with the pattern in the total network, mean and maximum values of the network measures were highest in the network of knowing each other.

**Table 3:** Description of individual networks (lowest and highest values per individual, mean between brackets)

	Knowing each other	Having professional contact
Size (one-step reach)	4 to 40 (17.4)	0 to 28 (8.9)

Number of connections (ties)	5 to 373 (123.0)	0 to 127 (28.5)

Density	11 to 96% (36.0)	0 to 100% (28.4)

Two step reach	34 to 99 (83.6)	0 to 84 (46.5)

Reach efficiency	12 to 77% (29.6)	0 to 100% (51.6)

Indegree centrality	2 to 39 (14.2)	0 to 23 (6.6)

Outdegree centrality	0 to 36 (14.2)	0 to 23 (6.6)

Incloseness centrality	23.2 to 38.0 (32.3)	1.0 to 9.7 (8.1)

Outcloseness centrality	1 to 58.5 (38.0)	1.0 to 12.7 (10.6)

Inreach centrality -2 steps	40 to 70 (53)	1 to 55 (36)

Outreach centrality -1 step	1 to 67 (53)	1 to 56 (36)

Betweenness centrality (normalized)	0 to 6.6 (1.1)	0 to 9.5 (1.6)

Size: number of individuals who are connected on one step to an individual, plus the individual.

Density: proportion of connections in an individual's network of connections of all possible connections which are present.

Two step reach: number of individuals that can be reached in 2 steps by an individual.

Reach efficiency: two step reach divided by network size. It indicates how efficient an individual network is with respect to reaching others in the total network.

Degree centrality. Number of (in/out) going connections of an individual. Individuals who receive many connections may be prominent or have high prestige, while individuals who connect to many others may be influential. The measure refers to direct connections to an individual only.

Closeness centrality. Distance of an individual to all others in the network (define by in/outgoing connections), here defined as the sum of the lengths of the shortest geodesic paths from an individual to others. The measure is standardized by norming against the minimum possible closeness in a network of the same size and connection.

Reach centrality. The number of individuals an individual can reach in a specific number of steps in the network of in/outgoing connections.

Betweenness centrality. Number of pathways in the network in which an individual is 'in between' of two other individuals. The measure indicates how frequently an individual is an intermediate between others. The maximum would be reached if the individual is the central person in a perfect star network.

Table 4 shows the same 12 measures in the predefined subgroups. Health professionals with ≥10 PD patients had higher mean and maximum values for 8 out of the 12 network measures. For one measure, reach efficiency, the difference was also significant but lower in professionals with ≥10 PD patients. No statistical difference was found for three measures: density, incloseness centrality, and outcloseness centrality. Regarding care setting, professionals in primary care had lower values on 11 of 12 measures compared to professionals who were (partly) based in hospital care. The measure for reach efficiency was significantly higher in primary care professionals.

**Table 4:** Individual networks by experience and setting of care delivery (lowest and highest values per individual, mean between brackets)

	Number of PD patients under treatment			Setting of care delivery			
	**≥10** **(n = 43)**	**< 10** **(n = 58)**	**P-value of difference**	**Primary (n = 35)**	**Hospital (n = 17)**	**Both** **(n = 51)**	**P-value of difference**

Size (one-step reach)	0 to 28 (11.4)	0 to 19 (7.1)	0.0003	0 to 20 (4.8)	4 to 28 (11.6)	0 to 22 (10.9)	0.0001

Number of connections (ties)	0 to 127 (41.0)	0 to 93 (19.2)	0.0003	0 to 69 (8.9)	5 to 127 (45.6)	0 to 93 (36.7)	0.0001

Density	0 to 73% (27.6)	0 to 100% (29.0)	0.7249	0 to 50% (16.3)	9 to 81% (36.9)	0 to 100% (32.7)	0.0001

Two step reach	0 to 84 (55.0)	0 to 78 (40.2)	0.0007	0 to 81 (30.5)	31 to 84 (56.4)	0 to 83 (54.5)	0.0001

Reach efficiency	0 to 97% (45.0)	0 to 100% (56.5)	0.0187	0 to 100% (67.7)	24 to 70% (42.9)	0 to 78% (43.1)	0.0001

Indegree centrality	0 to 23 (8.8)	0 to 15 (4.9)	0.0002	0 to 13 (3.4)	4 to 23 (9.4)	0 to 16 (7.9)	0.0001

Outdegree centrality	0 to 23 (8.5)	0 to 18 (5.2)	0.0011	0 to 20 (3.3)	4 to 23 (8.3)	0 to 22 (8.3)	0.0001

Incloseness centrality	1.0 to 9.7 (8.5)	1.0 to 9.3 (7.6)	0.0742	1.0 to 9.3 (7.3)	8.3 to 9.7 (8.8)	1.0 to 9.0 (8.5)	0.0014

Outcloseness centrality	1.0 to 12.7 (10.9)	1.0 to 12.7 (10.3)	0.3444	1.0 to 12.6 (9.4)	1.0 to 12.5 (10.4)	1.0 to 12.7 (11.4)	0.0034

Inreach centrality -2 steps	1 to 55 (40)	1 to 48 (33)	0.0004	1.0 to 43.4 (28.8)	32.8 to 54.6 (42.1)	1.0 to 49.7 (39.0)	0.0001

Outreach centrality -1 step	1 to 56 (39)	1 to 52 (34)	0.0530	1.0 to 54.3 (28.8)	1.0 54.9 (37.5)	1.0 to 56.4 (40.5)	0.0001

Betweenness centrality (normalized)	0 to 9.5 (2.4)	0.0 to 4.9 (1.0)	0.0002	0 to 7.3 (0.9)	0 to 9.5 (2.5)	0 to 7.2 (1.8)	0.0093

Legend: See Table 3.

Finally, the explorative factor analysis identified three factors with Eigen value > 1, which explained 86% of the variation of scores on 12 network measures across individuals. Network measures which load highly on the same factor correlate highly, which may reflect a shared underlying dimension. The first factor included network size, number of connections, two-step reach, reach efficiency, indegree centrality, outdegree centrality, and betweenness centrality (factor loadings > 0.75). The second factor included incloseness centrality, outcloseness centrality, inreach centrality, and outreach centrality (factor loadings > 0.73). The third factor included density (factor loading = 0.91).

## Discussion

This study examined the connectedness between health professionals involved in the treatment of patients with PD. The high participation rate and reasonably high reciprocity of reported connections suggests that the recruitment and the measure were feasible. In two of the three geographical sub-areas, we found higher values for network density and other network measures compared to the total network, suggesting that health professionals were more connected within their geographical area than in the total network. Measures related to individual networks of the health professionals showed a large variation. The number of patients treated per professional appeared to be an important determinant: health professionals with ≥10 PD patients yielded higher values on most network measures compared to those with < 10 PD patients, except for network density and in/outcloseness centrality. Primary care professionals yielded lower values for most network measures compared to professionals based in hospital settings. We conclude that the analysis of the network of health professionals showed relevant variation across individuals and geographical areas.

One strength of this study was the high participation rate, which may be related to the fact that completing the questionnaire was integrated in an educational meeting. ParkinsonNet provided a special context for this study. We should also mention several shortcomings. One weakness of our approach is the possibly limited generalizability of our findings, which may be restricted to health professionals who participate in a newly starting and disease-specific regional network. However, disease-specific networks have emerged in different clinical domains. A second limitation was that the measure of professional contacts was crude and not validated against a gold standard. However, it was straightforward and easy to understand. Third, the distinction between three geographic areas within the region was somewhat arbitrary for a few professionals. Finally, the factor analysis suggested that some network measures were highly correlated. As the network measures measure different constructs, this does not necessarily imply that measures with high correlation reflect some common underlying construct.

In a previous study we examined the communication and collaboration networks of 67 health professionals in 10 primary care practices regarding chronic heart failure, diabetes, and chronic obstructive pulmonary disease [16]. Using a short structured measure, we found good agreement between health professionals' reports on receiving and providing information. Networks measures for density and degree centralization showed large variation across practices, as did the degree of overlap between the three disease-specific networks. A difference with the current study is that our previous study focused on professional networks with primary care practices, while the current study examined a multidisciplinary network of health professionals in a region. Furthermore, ParkinsonNet is an innovative concept, while our previous study focused on usual primary care for chronic diseases.

We found that professionals who treated ≥10 PD patients were potentially more prominent and more influential in the network, as indicated by their higher indegree and outdegree centrality measures. This places them in a position to influence other health professionals, and thus spread professional competence in PD treatment and enhance the coordination of patient care. Notably, professionals with < 10 PD patients had density and closeness centrality measures that were similar to professionals with ≥10 PD patients. Network density may be related to acceptance and sanctioning of specific behaviors [14], so this would imply that the speed of uptake of new knowledge is not delayed by network characteristics. Primary care professionals were less connected in the network than professionals based in hospital settings. This finding should be interpreted in the context of the newly established network. One of the aims of ParkinsonNet is to better integrate primary care professionals in the treatment of patients with PD [8], so it would be interesting to repeat the study in a few years.

Network science provides a set of concepts and methods to study connectedness between elements in any system. Network approaches have been applied in many scientific disciplines, including neurosciences, molecular life sciences, and public health [17-19]. Its application in medical care research is relatively new, although the first use (concerning the uptake of new treatments by physicians) dates back to 1957 [20]. Examples in recent years include studies of opinion networks of long-term care specialists [21] and chronic disease networks in primary care [22]. In medical care research, network science offers the tools to conceptualize and measure specific network characteristics, which may be related to relevant outcomes. A social network approach may be particularly relevant if actors have imperfect information on their behavioral options and expected outcomes.

Communication and collaboration networks of health professionals reflect their communication and collaboration behaviors. At the same time, these network structures provide opportunities, incentives, and constraints for these individuals (and their patients). First, access to health professionals with relevant resources (such as clinical knowledge or ability to refer patients) may be influenced by the structure of networks. Second, many patient outcomes in chronic illness care can only be achieved if the clinical activities of different health professionals are intentionally coordinated. Third, a high degree of connectedness enhances imitation of behaviors and related social processes, resulting in more homogeneous practice patterns. Thus, whether a patient receives safe and effective treatment is not randomly distributed in a cohort of patients, but (*ceteris paribus*) more likely in networks with specific network measures.

Future research should focus on the development over time in networks of health professionals and on differences between networks in different regions. It should also focus on the impact of network measures on clinical treatment and outcomes. Future studies should also focus on the networks of individuals with chronic illness and include non-professionals who are relevant for their health and well-being [22]. Studies of networks in healthcare could provide relevant information for managers and policy makers in healthcare, if it would be clear how network characteristics are linked to relevant aspects of clinical treatment. For instance, individuals who have a central position in the network could be targeted in order to optimize the outcomes of professional networks such as ParkinsonNet. Like in other fields, a network approach promises to provide a new perspective on the coordination and delivery of healthcare.

## Competing interests

The authors declare that they have no competing interests. MW is an Associate Editor of Implementation Science. All decisions on this manuscript were made by another senior Editor. BB and MM initiated ParkinsonNet, which provided the context of the presented study.

## Authors' contributions

MW designed the study, was responsible for data analysis, and wrote the paper. ME was responsible for data collection and JK performed data analysis. All authors critical feedback and approved the final manuscript.
